# Social Determinants of Health and Cancer Survivorship

**Published:** 2021-08-14

**Authors:** Steven S. Coughlin

**Affiliations:** 1 Department of Population Health Sciences, Augusta University, 1120 15^th^ Street, Augusta, GA 30912; 2 Institute of Public and Preventive Health, Augusta University, Augusta, GA

## Introduction

### Statement of the problem

Recent research supports the premise of studying social determinants of health in conjunction with cancer survivorship (Hastert TA, 2021). It is widely appreciated that the social context in which people live and work influences their health (Coughlin SS, 2019).The World Health Organization defined the social determinants of health as the “conditions in which people are born, grow, work, live and age, and the wider set of forces and systems shaping the conditions of daily life” (WHO, 2014).

In recent years, increasing efforts have been made in health care settings to screen for a broad array of social determinants of health including inadequate food and nutrition and inadequate housing, and to refer patients to community resources (e.g., food pantries, housing programs) (Garg A, 2013; Gottlieb LM, 2015; Bazemore AW, 2016; Page-Reeves J, 2016; Pinto AD, 2016; DeVoe JE, 2016; Billioux A, 2017; Cottrell EK, 2018; LaForge K, 2018). According to this perspective, quality health care includes the amelioration of the harmful health effects of a lack of basic needs, and health care settings are an appropriate environment for assessing and intervening on social needs (social determinants of health) (Garg A, 2013; Gottlieb LM, 2015; Bazemore AW, 2016; Page-Reeves J, 2016; Pinto AD, 2016; DeVoe JE, 2016; Billioux A, 2017; Cottrell EK, 2018).

### Unemployment, lack of education, and low-income

Socioeconomic factors such as unemployment, lack of education, poverty, and income inequality are among the most important social determinants of health. It is well-established that low-income people are at increased risk of an array of adverse health outcomes and more likely to die prematurely. Numerous studies have documented a socioeconomic gradient: at each step along the socioeconomic ladder, there are improved health outcomes over the rung below (Kawachi I, 1999; Daniels N, 2000). In addition, the socioeconomic status gradient does not appear to be explained by differences in access to health care. Steep gradients have been observed even among groups of people who have adequate access to health care, housing, and transportation (Daniels N, 2000). There are identifiable pathways through which social inequalities appear to lead to health inequalities. In the United States, for example, states with the most unequal income distributions invest less in public education and spend less on social safety nets (Daniels N, 2000). Policies that improve individual life opportunities such as investment in basic education, affordable housing, and income security are likely to reduce health inequalities (Ruger JP, 2004).

There are well-documented disparities in cancer survival by socioeconomic status, race, education, poverty, and access to health insurance and medical care (Coughlin SS, 2019; 2020(a); 2020(b)). Poverty is associated with other factors related to poorer survival such as inadequate health insurance, lack of a primary care physician, and poor access to health care. To address these social determinants, effective interventions are needed that account for the social and environmental contexts in which cancer patients and cancer survivors live and are treated (Coughlin SS, 2019; 2020(a); 2020(b)).

### Housing Insecurity

There has been increasing awareness of the importance of social determinants of health such as access to safe and affordable housing in improving health outcomes among patients with cancer and cancer survivors. Although definitions vary, housing insecurity refers to a variety of housing experiences, including high housing costs in relation to income, frequent moves, and homelessness (Martin P, 2019). Housing insecurity affects millions of Americans with nearly 19 million households paying more than 50% of their income in housing costs (The State of the Nation’s Housing 2016; Martin P, 2019). Low-income, African American (AA), and unmarried adults, women, and younger adults are more likely to report housing insecurity (Charkhchi P, 2017; Martin P, 2019).

In January 2017, there were an estimated 553,742 homeless people in the United States (National Alliance to End Homelessness). The rate of people experiencing homelessness on a given night is about 17 per 10,000 people. Most homeless people lived in some form of shelter or in transitional housing. However, about 34 percent (192,875 people) lived in a place not meant for human habitation such as the street or an abandoned building.

People challenged by homelessness are living with several losses including the loss of a home, employment, economic security, health or well-being and personal security. For people who are homeless, assistance programs consist of housing, emergency shelter, food services, employment assistance, peer support, medical care, and mental health services including those aimed at recovery from substance-related disorders (Garg A, 2013; WHO, 2014). Such programs are administered by a variety of federal and state agencies, nongovernmental organizations, faith-based organizations, and veteran service organizations (Garg A, 2013; Gottlieb LM, 2015).

Cancer patients and cancer survivors may struggle to pay for housing or fall behind on their monthly bills to pay for out-of-pocket medical costs (Zafar SY, 2013; Zheng Z, 2020). Housing insecurity increases the likelihood of poorer health and decreased access to health care (Charkhchi P, 2017). Patients with housing insecurity and other unmet social needs have higher rates of chronic conditions such as depression, they are more likely to use the emergency department for care, and they are more likely to miss scheduled office visits (Cole MB, 2020). The financial burden of medical care as a cause of poorer health outcomes is worsened by other hardships such as the inability to afford housing (Charkhchi P, 2017).

The prevalence of home ownership is lower among AAs (47%) than among whites (76%), and AAs are almost 7 times as likely as whites to be evicted (Hastert TA, 2021). Relatively high levels of housing stability among AAs have negative effects on health, including anxiety, depression, hospitalization, and barriers to healthcare (Kushel MB, 2006; Burgard SA, 2012; Hastert TA, 2021).Providers who care for cancer survivors who are AA, low income, or have less educational attainment should be aware of the potential for housing insecurity and the potential for negative impacts on health outcomes. Other groups of cancer survivors who are at-risk of housing insecurity include women and those who are less than 65 years of age.

### Food Insecurity

Food insecurity is an important social determinant of health (Murthy VH, 2016) (28).The U.S. Department of Agriculture defines food insecurity as “a household-level economic and social condition of limited or uncertain access to adequate food” (US Department of Agriculture). Low-income, racial/ethnic minority and female-headed households are at greatest risk for food insecurity (US Department of Agriculture). People who experience food insecurity often consume a nutrient-poor diet, which may contribute to cancer risk factors such as obesity and diabetes (Seligman HK, 2010; Franklin B, 2012; Murthy VH, 2016). In order to buy food or because of budget constraints, low-income families may postpone medical care and underuse medicine (Murthy VH, 2016). Food insecurity is associated with stress, anxiety, depression and psychological distress (Bruening M, 2017).

In 2016, 12.3% of US households reported being food insecure at some point in the year (DeMarchis EH, 2019). The prevalence of food insecurity was 22.6% among non-Hispanic blacks and 31.6% in households headed by single women (DeMarchis EH, 2019). There are different stages of the severity of food insecurity starting with not being able to buy and eat what one would like due to income-related constraints. The next stage involves a decrease in food quantity, attempts to make food last until there is money to buy more, and hunger (Carter MA, 2013).

People who experience food insecurity often consume a nutrient-poor diet, which may contribute to obesity and other chronic conditions (Bruening M, 2017; Berger MH, 2020). In order to buy food or because of budget constraints, low-income families may postpone medical care and underuse medicine. Food insecurity is associated with stress, anxiety, depression and psychological distress (Carter MA, 2013; Zheng Z, 2020).

Federal programs to address food insecurity include the Supplemental Nutrition Assistance Program (SNAP), the Special Supplemental Nutrition Program for Women, Infants, and Children, the National School Lunch Program, the Child and Adult Care Food Program, and Meals on Wheels (Gualtieri MC, 2018).

An increasing number of studies have examined food insecurity among cancer patients and among cancer survivors who have completed primary therapy for the disease (Simmons LA, 2006; Gany F, 2014; Gany F, 2015; Bruening M, 2017; Charkhchi P, 2017; Bilodeau M, 2018; Trego ML, 2019; Berger MH, 2020; Hastert TA, 2020; McDougall JA, 2020). Many adverse effects associated with cancer and its treatment are also associated with food insecurity, including fatigue, depression, restricted activity, malnutrition, and weakened resistance to infection (Simmons LA, 2006). Food insecure patients may not comply with prescribed therapies because they may be choosing between paying for food or paying for medical care (Holben D, 2004; Simmons LA, 2006).

### Social Support and Social Network

Social support and social network is another key element of the social determinants of health. Presence of social network and high levels of social support have been shown to be a protective factor for maintaining good health and quality of life (Walker RJ, 2014; Bélanger E, 2016). Evidence showed social support was positively associated with physical and mental health, good self-rated health, reduced depression, and good quality of life, which are important indicators of overall well-being (Walker RJ, 2014; Bélanger E, 2016). In addition, social support and network play vital roles in patients’ navigating healthcare system and healthcare experiences (Gage-Bouchard EA, 2017). Findings show that patients who had adequate social support from their networks had more healthcare access, treatment options, more engaged to their care, more adhered to treatment regimens, fostered more productive relationships with their healthcare providers (Gage-Bouchard EA, 2017). Effective primary care demands patient/family’s time and attention to improve medical knowledge, communication skills, a proactive attitude to engage their self-care. Without adequate social support through their networks, it is impossible for patients having time and attention to build, refine, and leverage their ability navigating the health care system.

Among cancer patients, social isolation has been associated with poorer quality of life (Graells-Sans A, 2018). Presence of social network and high levels of social support have been shown to be a protective factor for maintaining good health and quality of life. For example, several studies have shown that cancer patients who are married have improved survival (Funch D, 1983; Marchand L, 1984; Goodwin J, 1987; Aizer AA, 2013; Parise C, 2018). Social support has been positively associated with physical and mental health, good self-rated health, reduced depression, and good quality of life, which are important indicators of overall well-being (Walker RJ, 2014; Bélanger E, 2016).

## Conclusions

When caring for cancer patients and cancer survivors, housing insecurity and food insecurity are important considerations for clinical oncology and primary care practice, especially when caring for patients with lower socioeconomic status and racial/ethnic minorities. Screening cancer patients and cancer survivors for housing insecurity, food insecurity, and financial distress and referring patients with social needs to community resources is likely to be beneficial. Additional studies are needed with a longitudinal design to examine the effectiveness of interventions aimed at addressing housing insecurity and food insecurity among cancer patients. Of particular interest are studies that focus on low-income AAs.
